# Data on the epidemiology, diagnosis, and treatment of patients with pneumothorax

**DOI:** 10.1016/j.dib.2018.08.063

**Published:** 2018-08-28

**Authors:** Manouchehr Aghajanzadeh, Mohammad Reza Asgary, Mohammad Sadegh Esmaili Delshad, Mahsa Hoseini Khotbehsora

**Affiliations:** Inflammatory Lung Disease Research Center, Razi Hospital, School of Medicine, Guilan University of Medical Science, Rasht, Iran

**Keywords:** Pneumothorax, Epidemiology, Traumatic pneumothorax

## Abstract

This data was acquired using a cross-sectional design in which medical records of patients admitted at Arya and Razi Hospitals of Rasht from 2006 to 2015 were examined. The patients’ demographic data, history of smoking and opium, underlying disease, clinical symptoms at admission, the utilized diagnostic method, duration of hospitalization, findings of chest CT scan, type of pneumothorax, and therapeutic technique were collected through a questionnaire. The collected data were encoded and analyzed using SPSS 21.0. Smoking rate was measured in the primary and secondary spontaneous pneumothorax groups and acquired pneumothorax group. The most frequent underlying disease in the patients with secondary spontaneous pneumothorax (SSP) was COPD that was observed in 41 patients (51.25%). The frequency of bleb was measured in the three groups. Out of 38 patients (15.01%) with recurrence of pneumothorax, 68.42% had PSP type. Chest tube was the most frequently used therapeutic technique, which was utilized 92.88% out of 235 patients.

## Specifications Table

**Table:** 

Subject area	Medicine
More specific subject area	Respiratory diseases
Type of data	Tables
How data was acquired	This data was acquired from medical records of patients admitted at Arya and Razi Hospitals of Rasht that were examined from 2006 to 2015.
Data format	Raw and analyzed
Experimental factors	Patients were categorized into three groups: primary spontaneous pneumothorax, secondary spontaneous pneumothorax, and with acquired pneumothorax.
Experimental features	Data collection instrument included a researcher-designed questionnaire that consisted of the patients’ demographic data including age and gender, history of smoking and opium, underlying disease (tuberculosis, chronic obstructive pulmonary disease, cystic fibrosis, lung cancer, and asthma), clinical symptoms at admission, the utilized diagnostic method, duration of hospitalization, findings of chest CT scan, type of pneumothorax, and therapeutic techniques.
Data source location	Rasht, Guilan province, Iran
Data accessibility	Data are included in this article
Related research article	D. Gupta, A. Hansell, T. Nichols, T. Duong, J.G. Ayres, D. Strachan, Epidemiology of pneumothorax in England, Thorax. 55 (2000) 666–671 [1].

## Value of the data

• This data can be useful for researchers investigating the epidemiological characteristics of pneumothorax including prevalence of risk factors, underlying disease, and prevalence in age groups and both sexes.
• This data provides interesting information on the epidemiological status of pneumothorax and its association with smoking and presence of blebs and bullae in HRCT.
• The data included in this article can be important because they are different from the previous evidences.

## 1 Data

Out of 253 patients, 45 patients (17.79%) were women and 208 patients (82.21%) were men. In addition, 116 patients (45.8%) were diagnosed with primary spontaneous pneumothorax, 80 patients (31.6%) with secondary spontaneous pneumothorax, and 57 (22.5%) with acquired pneumothorax. Table 1 shows the frequency of symptoms among the patients based on the different types of pneumothorax. In addition, Table 2 presents the radiography findings including bullae, bleb, and emphysema in the three types of pneumothorax.

**Table 1:** The frequency of symptoms among the patients based on different types of pneumothorax.

**Pneumothorax type**	**Chest pain**	**Decrease in tactile fremitus**	**Hyperresonance**	**Shortness of breath**
	**Frequency (%)**	**Frequency (%)**	**Frequency (%)**	**Frequency (%)**
PSP	99 (85.34%)	68 (58.62%)	15 (12.93%)	107 (92.24%)
SSP	60 (75%)	49 (61.25%)	8 (8.75%)	78 (97.5%)
PSP	99 (85.34%)	68 (58.62%)	15 (12.93%)	107 (92.24%)
Acquired	49 (85.96%)	27 (47.36%)	9 (15.93%)	54 (94.73%)
SSP	60 (75%)	49 (61.25%)	8 (8.75%)	78 (97.5%)
Acquired	49 (85.96%)	27 (47.36%)	9 (15.93%)	54 (94.73%)

**Table 2:** The radiography findings including bullae, bleb, and emphysema in the three types of pneumothorax.

**Type of pneumothorax**	**Emphysema**	**Bleb**	**Bullae**
	**Frequency (%)**	**Frequency (%)**	**Frequency (%)**
PSP	4 (3.4%)	32 (27.5%)	20 (17.2%)
SSP	7 (8.75%)	1 (1.25%)	24 (30%)
PSP	4 (3.4%)	32 (27.5%)	20 (17.2%)
Acquired	10 (17.5%)	0 (0%)	0 (0%)
SSP	7 (8.75%)	1 (1.25%)	24 (30%)
Acquired	10 (17.5%)	0 (0%)	0 (0%)

## 2 Experimental design, materials and methods

This data was obtained using a cross-sectional design from all hospitalized patients (253 patients; 45 (17.79%) women and 208 (82.21%) men) in Arya and Razi Hospitals of Rasht during 2006–2015. Inclusion criterion was all patients diagnosed with pneumothorax. The exclusion criteria included incompleteness of the patients’ records and accompany of other pleural space diseases (pleural effusion, empyema, and hemothorax).

After necessary permissions were obtained from the Research Department of Guilan University of Medical Sciences, the qualified records of all patients admitted in Arya and Razi Hospitals of Rasht were examined with the help of the hospitals’ administration.

Data collection instrument included a researcher-designed questionnaire that consisted of the patients’ demographic data including age and gender, history of smoking and opium, underlying disease (tuberculosis, chronic obstructive pulmonary disease, cystic fibrosis, lung cancer, and asthma), clinical symptoms at admission (chest pain, shortness of breath, irritability of the airways, and epigastric pain), the utilized diagnostic method (chest x-ray radiography and chest CT scan), duration of hospitalization, findings of chest CT scan, type of pneumothorax (spontaneous, iatrogenic, and traumatic), and therapeutic technique (supportive therapy, simple aspiration, chest tube placement, thoracotomy, pleurodesis, video-assisted thoracoscopic surgery (VATS)).

The data was extracted from the questionnaires and entered to a Microsoft Excel spreadsheet (Microsoft Office 2016). The collected data were first encoded then analyzed using SPSS 21.0. The number and frequency of different variables were calculated. Patients were categorized into three groups: primary spontaneous pneumothorax, secondary spontaneous pneumothorax, and with acquired pneumothorax.
